# Stimulus Repetition and the Perception of Time: The Effects of Prior Exposure on Temporal Discrimination, Judgment, and Production

**DOI:** 10.1371/journal.pone.0019815

**Published:** 2011-05-09

**Authors:** William J. Matthews

**Affiliations:** Department of Psychology, University of Essex, Colchester, United Kingdom; Duke University, United States of America

## Abstract

It has been suggested that repeated stimuli have shorter subjective duration than novel items, perhaps because of a reduction in the neural response to repeated presentations of the same object. Five experiments investigated the effects of repetition on time perception and found further evidence that immediate repetition reduces apparent duration, consistent with the idea that subjective duration is partly based on neural coding efficiency. In addition, the experiments found (a) no effect of repetition on the precision of temporal discrimination, (b) that the effects of repetition disappeared when there was a modest lag between presentations, (c) that, across participants, the size of the repetition effect correlated with temporal discrimination, and (d) that the effects of repetition suggested by a temporal production task were the opposite of those suggested by temporal judgments. The theoretical and practical implications of these results are discussed.

## Introduction

The judgment of time is central to many behaviours. Learning patterns of reinforcement [1], interpreting communication signals [2], [3], and encoding dynamic stimuli [4]–[7] all rely upon the accurate representation of temporal information. Despite the importance of accurate timing, time perception is influenced by various non-temporal factors, including intensity [8]–[10], motion [11]–[13], and sensory modality [14]–[16]. These effects have been interpreted within various theoretical frameworks, including those which posit some kind of internal pacemaker or counting process [17]–[19], attentional models [20], [21], and the idea that time perception is based upon the magnitude or unfolding pattern of neural activity triggered by a stimulus [22], [23]. The current work examined the effects of stimulus repetition on temporal judgment, and addressed five empirical questions.

1. Does immediate repetition shorten perceived duration? Existing data suggest that subjective duration is reduced for stimuli which have just been encountered. Evidence for this comes from experiments in which a standard stimulus is repeated approximately 8–10 times with a single presentation of a different “oddball” stimulus near the end of the sequence. The oddball is typically judged to last longer than standard stimuli of the same physical duration (although the effect may reverse for very short durations) [24]–[26]. Similarly, the first image in a sequence is judged longer than the others when the stimuli are identical but not when they are different [25], and the visual persistence of briefly-flashed images is reduced for repeated items [27]. These results have been taken as evidence that subjective duration depends on the size of the neural response to a stimulus [22], [25], because repeated stimuli evoke smaller responses (“repetition suppression” [28], [29]). The current experiments provide further data regarding the effects of repetition on the apparent duration of stimuli lasting several hundred milliseconds.
2. Does immediate repetition influence the precision of temporal discrimination? Discrimination involves comparing the current stimulus with a standard encoded in memory. The subjective duration of a stimulus depends on its non-temporal properties, so there may be more noise in the comparison of the current duration with the memory standard when the two intervals are demarcated by different visual stimuli, leading to poorer discrimination. More generally, different theoretical accounts of time perception posit differing effects of prior exposure on discrimination accuracy and subjective duration [30], [31] but relevant data are in short supply. In one study, Ulrich et al. found that the comparison duration was subjectively longer and the discrimination more precise if the comparison stimulus was rare than if it was common [30], although it is unclear whether this is because the common stimuli were presented more frequently, more recently, or both.
3. How are the effects of repetition influenced by changes in lag? Previous work suggests that when the repetition lag is several minutes, prior exposure increases subjective duration – the opposite of what has been found when repetition is immediate. For example, Witherspoon and Allan found that briefly presented words (c. 50 ms) were judged to last longer when they had recently been read aloud [32]. Similarly, participants asked to leave a stimulus on-screen for 2.5 seconds waited less time when the stimuli were studied words than when they were novel ones [33]. Temporal production is assumed to have an inverse relationship to estimated duration, so these data further suggest that the subjective duration of items encoded a few minutes previously is greater than that of unstudied stimuli. It is currently unclear whether the difference between these results and those obtained with immediate repetition are due to differences in the lag between presentations or to differences in the judgment tasks employed by the respective studies.
4. Is the extent to which an observer is influenced by stimulus repetition related to the accuracy of his or her temporal discrimination? Although there have been studies of individual differences in time perception (e.g., [34], [35]), differences in susceptibility to factors which affect subjective duration have not been much investigated.
5. Do the effects of repetition depend on the temporal judgment task? Different judgment tasks are often assumed to provide equivalent measures of the time percept, with well-established relationships between tasks. It is worth testing this assumption by using different judgment tasks to gauge the effects of stimulus repetition on subjective duration [24].

## Results

### Experiment 1: Temporal discrimination

**Figure 1:**
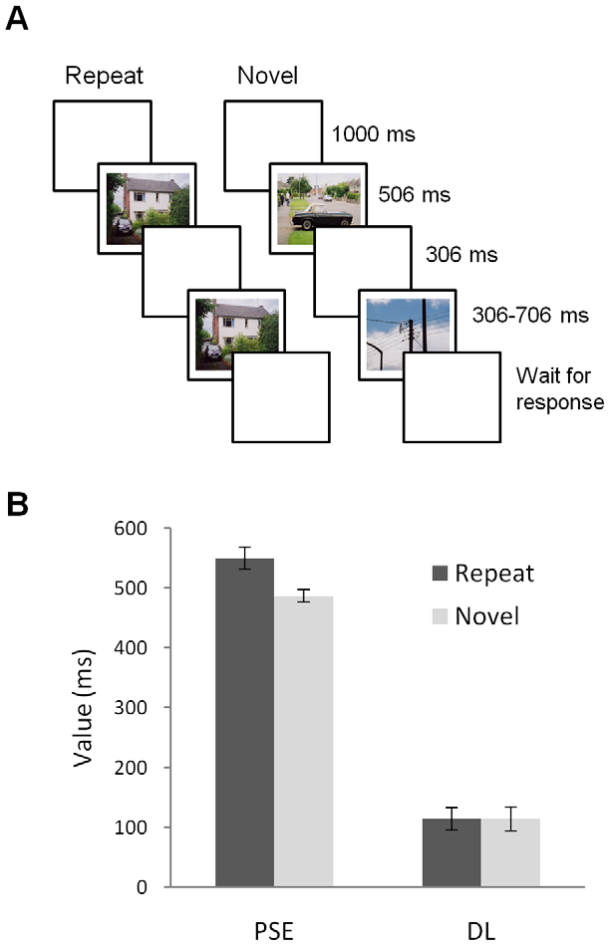
Trial structure and results of Experiment 1. Panel A shows the events on each trial, which began with a blank screen for 1000 ms. Participants judged whether the second image was shown for more or less time than the first. On repeat trials, the images were identical; on novel trials the images were different. Panel B shows the results. The point of subjective equality (PSE) was greater for repeated stimuli, indicating shorter subjective duration; repetition produced no discernible effect on the difference limen (DL), a measure of temporal discrimination. Error bars show plus/minus one SEM, calculated separately for each data point. Note that for a within-subject design such as this, these error bars provide no indication of the significance of differences between conditions [51].

Participants judged whether the duration of a comparison stimulus (306–706 ms) was longer or shorter than that of a standard stimulus (506 ms) (Figure 1A). On *repeat* trials, the comparison stimulus was identical to the standard; on *novel* trials, the comparison stimulus was a new picture, not previously encountered. Separate logistic functions were fit to the data from each participant and used to estimate the point of subjective equality (PSE: the comparison duration judged equal to the standard) and the difference limen (DL: a measure of the precision of temporal discrimination). Larger PSE values indicate shorter subjective durations for the comparison stimulus; larger DL values indicate poorer temporal discrimination.

The PSE was larger in the repetition condition than in the novel condition, *t*(13) = 3.05, *p* = .009 (Figure 1B). Thus, subjective duration was shorter for repeated items than for novel ones. Stimulus repetition had no appreciable effect on the difference limen, *t*(13) = .01, *p* = .99. That is, there was no effect of repetition on the precision of temporal discrimination.

### Experiment 2: Delayed repetitions

Experiment 2 was like Experiment 1 but introduced a *delayed repetition* condition in which the comparison image had served as the standard 21 trials previously, so that 20 trials and 42 images intervened between the two presentations of the picture. (The length of the inter-presentation interval depended on the participant's response times; average intervals ranged from 63.3–89.2 s, mean 74.9 , SD = 6.9 s).

**Figure 2:**
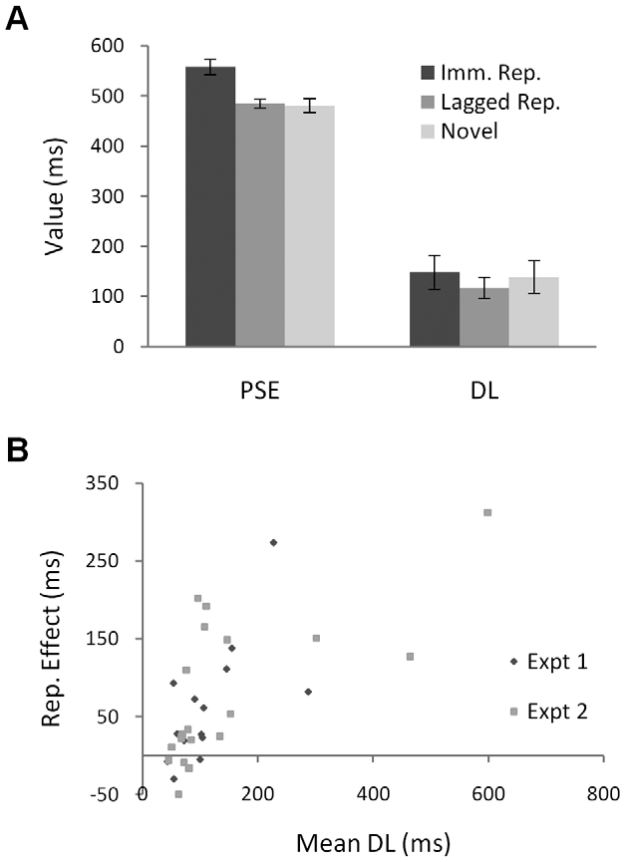
Results of Experiment 2. Panel A shows that the subjective duration was longer (the point of subjective equality was shorter) for novel images and lagged repeats (Lagged Rep.) than for immediate repeats (Imm. Rep); lagged repeats and novel images did not differ. The difference limen (the precision of temporal discrimination) did not differ between conditions. Panel B shows the positive across-participant correlation between temporal discrimination and the size of the repetition effect (the difference between the PSE for immediate repetitions and the PSE for novel stimuli). The pattern remained when the outlying participants with very poor discrimination were excluded. Error bars show plus/minus one SEM, calculated separately for each data point; these provide no indication of the significance of differences between conditions.

The results are shown in Figure 2. The point of subjective equality depended on the experimental condition, *F*(1.42, 27.02) = 12.66, *p*<.001, 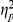 = .40 (Huynh-Feldt correction applied); immediate repeats seemed shorter than both delayed repeats and novel images, which did not differ (Bonferroni-corrected *p*s = .005, .003, and 1.000 respectively). The difference limen was unaffected by condition, *F*(2,38) = 2.63, *p* = .085, 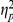 = .12.

Including data from both Experiments 1 and 2 in a single ANOVA indicated neither a main effect of experiment nor any interaction between experiment and condition (all *F*s<1). Collapsing over experiment replicated the pattern from the individual experiments: the subjective durations of novel stimuli were longer than those of immediate repeats, *t*(33) = 4.83, *p*<.001, but the difference limens were unaffected by repetition, *t*(33) = .73, *p* = .469. In this analysis, the power to detect a “medium” effect (*d = *0.5) in the difference limens is approximately 81% [36].

Across participants, the extent to which stimulus repetition influenced subjective duration was related to the fidelity of temporal discrimination. For each participant, overall temporal discrimination was indexed by averaging the difference limens across conditions. The size of the effect of repetition on subjective duration was indexed by subtracting the PSE for immediate repetitions from the PSE for novel stimuli. The results are plotted in Figure 2B. In both experiments there is a positive correlation between difference limen and the magnitude of the repetition effect (for Experiment 1, *r* = .65, *p* = .012, Spearman's rho = .64, *p* = .014; for Experiment 2, *r* = .64, *p* = .002, Spearman's rho = .71, *p*<.001). It is clear in Figure 2B that some participants show very poor discrimination; removing outlying participants with mean difference limens more than 1.5 interquartile ranges above the upper quartile (1 person in Experiment 1, 3 people in Experiment 2) showed that the correlations were not just driven by these extreme data points (for Experiment 1, *r = *.86, *p*<.001, Spearman's rho = .65, *p = *.017; for Experiment 2, *r = *.37, *p* = .142, rho = .62, *p* = .008; the *t*-test and ANOVA results reported above remained unchanged for these restricted datasets).

Thus, the difference between the subjective duration of repeated and novel stimuli was greater for participants with poorer temporal discrimination. This pattern remained when, rather than averaging the difference limen across conditions, the correlations were calculated separately using the DLs for immediate repeats (Experiment 1: *r* = .58, *p* = .030, rho = .69, *p* = .006; Experiment 2: *r* = .69, *p* = .001, rho = .80, *p* <.001) and novel images (Experiment 1: *r* = .66, *p* = .010, rho = .46, *p* = .095; Experiment 2: *r = *.65, *p = *.002, rho = .57, *p* = .009).

### Experiments 3A and 3B: Alternative judgment tasks

**Figure 3:**
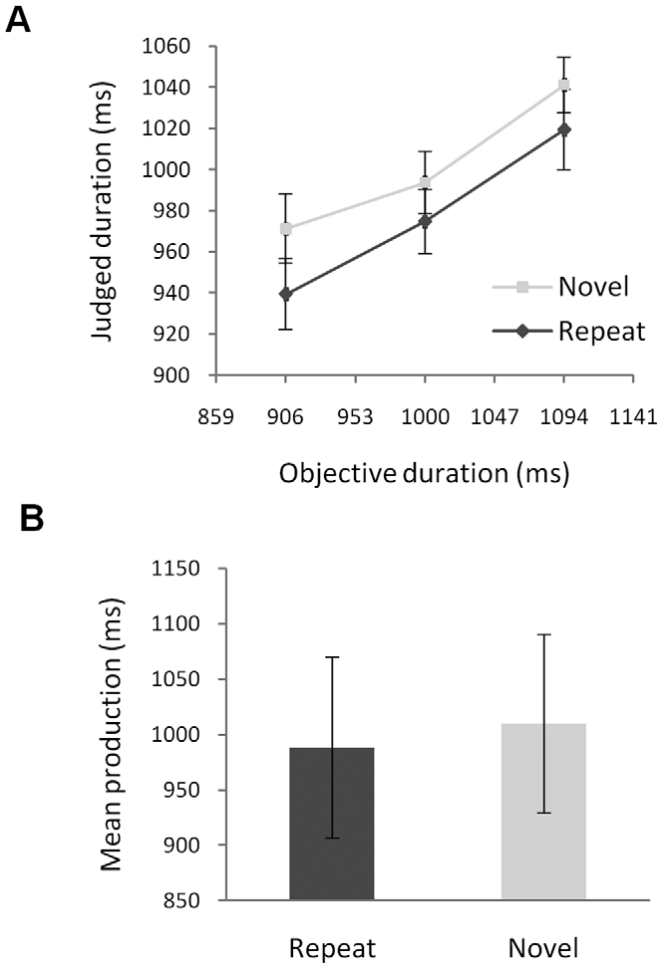
Results of Experiments 3A and 3B. Panel A shows the results of Experiment 3A, which used category judgment. Novel stimuli were judged to last longer than immediate repeats. Panel B shows the results of a temporal production task. Productions were longer for the novel images, which would usually be taken to indicate *shorter* subjective duration – in contradistinction to the results from the category judgment paradigm. The discrepancy between Panels A and B suggests that temporal production may be a poor index of subjective duration. Error bars show plus/minus one SEM, calculated separately for each data point. Again, these provide no indication of the significance of differences between conditions.

Experiments 3A and 3B were similar to Experiments 1 and 2, but used different judgment tasks. Experiment 3A used category judgment: the second picture on each trial was shown for 906, 1000 or 1094 ms and participants classified its duration on a scale ranging from 0.7–1.3 seconds in 0.1 s increments (Figure 3A). Judged durations increased with physical duration, *F*(2,42) = 25.69, *p*<.001, 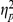 = .55. More importantly, repeated stimuli were judged shorter than novel ones, *F*(1,21) = 5.94, *p* = .024, 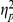 = .22; this effect did not interact with duration, *F*(2,42) = .67, *p* = .517, 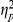 = .031.

Experiment 3B used temporal production: the second picture displayed until the participant pressed a button, and participants sought to leave the picture on-screen for 1 second (Figure 3B). Mean produced durations were longer for novel stimuli than for repeats, *t*(19) = 2.49, *p* = .022.

Repeated stimuli therefore produced shorter category judgments but also led to shorter mean temporal productions. This is surprising because temporal estimation and temporal production are widely believed to be inversely related [37], [38], [39]. As Brown (1997, p.1121) explains, for estimation tasks like Experiment 3A: “If conditions during the interval cause a reduction in the number of temporal cues perceived, subjects may be biased to judge the interval as having been relatively short” whereas for production tasks like Experiment 3B: “If the prevailing task conditions reduce the number or salience of temporal cues, then the subject may allow a relatively longer amount of time to pass by before he or she judged that the interval has elapsed” [37]. That is, given that stimulus repetition led to smaller judgments in Experiment 3A it would be expected to lengthen productions in Experiment 3B, contrary to what was found.

### Experiment 4: Further investigation of temporal production

What might underlie the surprising results of Experiments 3A and 3B? One possibility is that the effect of stimulus repetition on the rate or salience of temporal cues depends on the observer's task. For example, if time perception is based on the accumulation of pulses from an internal pacemaker then the pacemaker may run faster for novel items than repeats during discrimination or category judgment tasks (like Experiments 1–3A) but slower for novel items during productions tasks like (Experiment 3B). It is hard to see why pacemaker rate should depend on experimental task in this way, but the account nonetheless predicts that the effect of repetition on temporal production will become more pronounced as the to-be-produced duration increases, because the total number of accumulated pulses depends on the product of the pacemaker rate and the physical time interval [14], [38], [40].

In Experiment 4, participants completed a production task where the target duration was 0.5, 0.75, 1.0, 1.25 or 1.5 seconds, with one block of 30 trials per duration. As before, participants saw two pictures on each trial and had to press a button when the second picture had been on-screen for the specified amount of time. On half of the trials the two pictures were identical and on half they were different.

**Figure 4:**
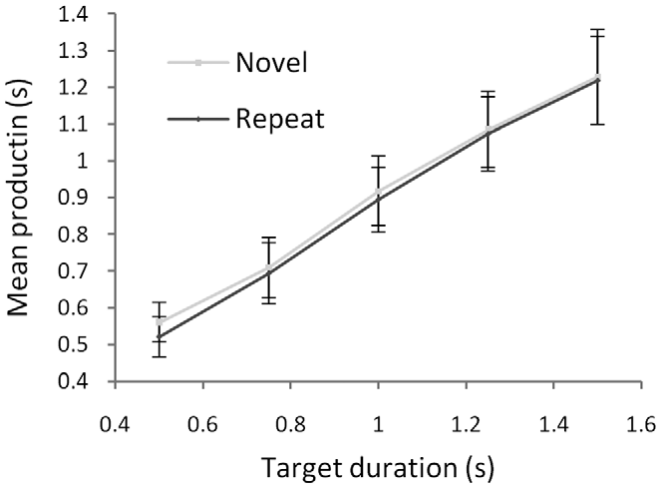
Results of Experiment 4. Mean temporal productions were longer for novel stimuli, which would usually be taken to indicate shorter subjective duration for these items – contrary to the results of Experiments 1, 2, and 3A. If the difference between novel stimuli and repeats reflected a difference in the rate of an internal pacemaker then the effect would become more pronounced at longer durations. In fact, the effect of repetition was independent of duration – and inspection of the figure suggests that, if anything, the difference between conditions is smaller at longer durations. Error bars show plus/minus one SEM, calculated separately for each data point. Again, these provide no indication of the significance of differences between conditions.

The results are shown in Figure 4. Temporal productions increased with increasing target duration, *F*(2.66, 69.03) = 36.09, *p*<.001, 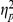 = .58. More importantly, temporal productions were shorter for repeated stimuli than for novel ones, *F*(1,26) = 5.87, *p* = .023, 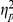 = .18, and this effect was independent of target duration, *F*(3.42, 88.96) = .50, *p* = .711, 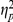 = .02. Thus, there was no evidence that repetition affected the slope of the production function in the manner predicted by a change in the rate of an internal pacemaker. The basis for the longer productions with novel stimuli is discussed in more detail below.

Experiment 4 also addressed a question posed by the results of Experiment 2: did delayed repetition have no effect on duration judgments because participants failed to recognize repeated items? After completing the production task of Experiment 4, participants were given a surprise recognition memory test in which they saw a mixture of novel images and “Old” pictures which had been seen during the production phase, and had to identify those which had been seen before. All recognition test images were shown for 506 ms. Old images were stimuli which had been presented as the first picture of each pair shown on non-repetition trials during the first 4 blocks of the production task. Each Old picture was therefore shown for 506 ms and had been seen precisely once for 506 ms at least one block of trials previously, making them similar to the delayed repetition stimuli in Experiment 2.

For each participant, the proportion of correct responses to Old pictures and New pictures were used to calculate d' as a measure of recognition accuracy. The mean d' was 1.00 (SD = 0.49), significantly above chance, *t*(26) = 10.55, *p*<.001. It is therefore likely that the delayed repetition stimuli in Experiment 2 would have been recognized with above-chance accuracy, suggesting a dissociation between the effects of prior exposure on overt recognition and the effects on temporal perception.

## Discussion

These results suggest several conclusions. First, Experiments 1 and 2 indicate that stimulus repetition reduces subjective duration. This replicates findings from the oddball paradigm [24]–[26] in a task where the participant is not required to judge the comparison stimulus against a large number of preceding items, and where the novelty of the item is not confounded with its position in the stimulus sequence. It accords with the idea that subjective duration correlates with the size of the evoked neural response [22], [25], [41], although the current data do not provide direct evidence of either the effects of repetition on neural activity or of the correlation between this activity and subjective time (but see [42]).

Second, the effect of repetition seems to be short-lived. In Experiment 2 there was no effect of having seen a stimulus a minute or two previously, despite the finding in Experiment 4 that participants could readily distinguish delayed repetitions from novel stimuli in an explicit memory test. Priming effects in other paradigms also diminish with lag [43], but it is not clear why the effect of prior exposure on temporal perception disappeared after only a short inter-stimulus interval filled with approximately 20 intervening images, particularly since previous studies have demonstrated memory effects on temporal judgment using comparable delays [32], [33], [44] – although these studies used judgment tasks and stimulus types which differ from those of Experiment 2. The precise time course of repetition effects on judgments of duration, and the question of whether these effects diminish over time, over intervening items, or both, will be important indicators of their neural basis [43].

Third, there was no indication that immediate repetition influences the precision of temporal discrimination. That is, repetition shortened subjective duration but had little effect on temporal sensitivity. Ulrich et al. examined the effects of whether a stimulus was expected or unexpected on temporal perception, manipulating expectations by presenting some items with high frequency and others with low frequency [30]. These authors discuss different possible effects of expectancy, and suggest that the pattern found in the current experiment would arise if expected (repeated) stimuli are processed especially quickly. Such an increase in processing speed would further accord with the idea that repetition improves the efficiency of neural coding, but speed of processing was not directly assessed here. It is also possible that the failure to find an effect on discriminability arose from noise in the estimates of the difference limen.

Fourth, across participants the difference between the subjective duration of repeated and novel items was related to the accuracy of discrimination. Although many “temporal illusions” have been documented [41] there has been little or no attention to individual differences in susceptibility to the factors which influence subjective duration. One possibility is that the more difficult participants found the discrimination (or the less they engaged with the task), the more they fell back on the use of non-temporal information as the basis for their judgments. Human judgment often involves a strategy of “attribute substitution” in which a difficult judgment is simplified by substituting a dimension that is correlated with the target attribute but easier to assess [45]. For example, the size of the neural response to a stimulus may be correlated with its physical duration, but will also depend on non-temporal factors – such as repetition. The magnitude of the neural response (that is, the coding efficiency) may therefore provide a reasonable basis for temporal judgment, but one that is susceptible to distortion if the observer does not attend to other relevant duration cues – such as the ongoing neural activity evoked by the other stimuli in the environment. To the extent that an observer is willing or able to integrate various different indices of duration, their judgments will be more accurate and less influenced by non-temporal factors such as repetition. It will be important to see whether the correlations found here replicate for other factors which bias duration judgments, such as intensity and modality.

Finally, the data show that the effects of repetition depend upon the temporal judgment task. In discrimination and category judgment tasks, repeated stimuli were judged shorter than novel ones. However, mean temporal productions were longer for novel stimuli than for repeats (Experiments 3B and 4; this effect was also replicated in an additional study not reported here). As described above, it is widely believed that temporal production bears an inverse relation to subjective duration: manipulations which slow the accumulation of temporal cues (for example, by reducing the rate of an internal pacemaker) are expected to shorten the perceived duration of a given physical interval and to increase the physical time needed to reach a given subjective duration (e.g., [33]) and several previous investigations have reported this reciprocal pattern [38], [39]. However, the dissociation between judgment and production in Experiments 3A and 3B argue against acceptance of this relationship as a universal principle.

What mechanism might underlie the finding that mean productions were longer for novel stimuli than repeats? Experiment 4 found no evidence that the size of the effect increased with increasing target duration in the manner predicted by an increase in the rate of an internal pacemaker. Stimulus repetition may therefore affect other aspects of the production task. Unlike discrimination and estimations tasks, temporal production requires that the observer continuously compare the current duration against a reference memory, with a match initiating a response which terminates stimulus presentation. One tentative explanation for the results of Experiments 3B and 4 is that it takes longer to disengage from viewing novel images in order to make a response when the target duration has been reached – for example, because viewing novel stimuli is more interesting than viewing repeats – leading to longer temporal productions. If there is a constant “disengagement latency” for novel stimuli, the effect of repetition on temporal production will be independent of the to-be-timed duration – as was found in Experiment 4. Such an effect would be separate from any influence of repetition on subjective duration *per se*, and would not affect judgments in discrimination or estimation tasks like those of Experiments 1, 2, and 3A.

This suggestion is speculative, but the dissociation between production and estimation/discrimination judgments nonetheless has important implications. Some studies of time perception – including research concerned with the effects of prior exposure on temporal perception – use only the production task to assess subjective duration [33], [44]. However, the current results urge caution about using temporal production as an index of subjective duration in this context. Similarly, Droit-Volet has recently warned about temporal production techniques, emphasizing the contribution of motor factors to performance [46], and there is evidence that temporal productions are affected by the events immediately prior to the production interval [11]. In contrast, simply asking people how long they think a stimulus lasted has been described as a the “rawest” type of temporal judgment [16], [47], and may be a better measure. More generally, it is worth noting the basic point that in the many experiments which seek to study time perception, the observer's percept is never directly observable. All such experiments involve a judgment task, and the nature of this judgment can shape our inferences about the neural or algorithmic bases for perception [40].

## Methods

### Ethics statement

Participants gave written informed consent. Ethical approval for all experiments was granted by the ethics committee of the University of Essex Faculty of Science and Engineering.

### Stimuli

In all experiments the stimuli were a set of 1200 landscape photographs (343×245 pixels) drawn from diverse sources such that participants were very unlikely to have encountered the images before. A random subset of images was chosen for each participant. Pictures were presented on 19″ CRT monitors (1024×768 pixels, 85 Hz). In Experiment 1 stimuli were presented against a black background and viewed from approximately 40 cm in quiet testing rooms (stimuli subtended approximately 17×12 degrees visual angle); in the other experiments stimuli were presented against a white background and viewed from approximately 60 cm through the glass walls of sound attenuating chambers (stimuli subtended approximately 11×8 degrees visual angle). Responses were made via computer keyboard and stimulus presentation was controlled by DMDX [48]; occasional trials were excluded because of display timing errors.

### Experiment 1

Fifteen participants took part for course credit or a payment of £4. On each trial, participants saw two pictures. The first, standard stimulus was always presented for 506 ms; the second, comparison stimulus was presented for 306, 353, 400, 447, 506, 565, 612, 659, or 706 ms (each comparison stimulus occurred equally often; intervals were chosen to be an integer number of screen refreshes). After offset of the second picture participants used response keys to indicate whether the second stimulus was longer or shorter than the first. The sequence of events on each trial is shown in Figure 1A.

On half of the trials, the same picture was used as both the standard and comparison stimulus (repeat trials); on the other half, the comparison picture was different from the standard (novel trials). Trial order was randomized. Different pictures were used on every trial. Participants completed 396 trials with the opportunity to pause every 12 trials.

For each participant, logistic psychometric functions were fit for each condition and used to calculate the point of subjective equality and difference limen. Fitting was performed with the lrm function of the Design package of R [49], [50]. One participant was excluded because the data deviated significantly from the logistic function, leaving 14 participants.

### Experiment 2

Twenty eight new participants completed the experiment for course credit. The task was similar to Experiment 1. Seven comparison durations were used (294, 365, 435, 506, 576, 647, and 718 ms). There were 25 blocks of 21 trials. The standard was a different randomly-chosen picture on every trial. On novel trials, the comparison picture was a different image not previously seen in the experiment; on immediate repetition trials, the comparison picture was identical to the standard; on delayed repetition trials, the comparison picture was the standard image shown 21 trials previously; this could have been a novel trial or a delayed repetition trial, but never an immediate repetition trial. Thus, the comparison pictures on both immediate repetition and delayed repetition trials had both been seen precisely once before, for 506 ms. The sequence of events on each trial was: a randomly-chosen blank interval for 882–1117 ms; the standard picture for 506 ms; a blank interval for 3061 ms; the comparison picture for 294–718 ms; and a blank interval until the participant responded.

Each block comprised one occurrence of each comparison duration in each experimental condition, in random order (subject to the constraint that the delayed repetition trials could not occupy the position in the block taken by the immediate repetition trials of the previous block, to ensure that delayed repeat pictures had been seen only once before.) The first block served as run in; no lag trials were possible, so there were two sets of novel trials and one set of immediate repetition trials. Participants were invited to take a break at the end of each block.

For each participant, logistic functions were fitted to the data from each condition. For eight participants the data deviated significantly from the logistic function for one or more of the three conditions, so these participants were excluded (their inclusion made no difference to the results).

### Experiments 3A and 3B

Forty three new participants took part for a payment of £3; alternating participants were assigned to the judgment task (Experiment 3A) and production task (Experiment 3B). One participant was excluded from the production task for failing to follow instructions, leaving 22 who completed the categorization task and 20 who completed the production task.

The sequence of events on each trial of the categorization task was: blank interval for 882–1059 ms; first stimulus for 506 ms; blank interval for 306–424 ms; the second stimulus 906, 1000 or 1094 ms; blank interval until response. Seven response buttons were labelled 0.7–1.3 seconds in 0.1 second steps; participants were told to press the button corresponding to the duration closest to the length of time that the second picture was on the screen. On half of the trials the second stimulus was identical to the first (repetition condition); on half it was different (novel condition). Each block of 18 trials comprised 3 occurrences of each condition-duration combination in random order. Participants completed 7 blocks; the first block was treated as practice and discarded from analysis.

The production task was identical except that the second stimulus remained on-screen until the participant tapped a key; participants were told to tap the key after the second picture had displayed for 1 second, and to rely on their intuitive sense of time rather than counting. Participants completed 7 blocks of 18 trials with 9 repetition and 9 novel trials per block in random order. Productions shorter than 200 ms or longer than 2500 ms were excluded as errors; their inclusion did not affect the pattern of significant results.

### Experiment 4

Twenty nine new participants took part for course credit or a payment of £3. Two were excluded (one failed to follow instructions; one produced extreme responses on more than 20% of trials).

The production task was identical to Experiment 3B. Participants saw two pictures on each trial; the first image stayed on-screen for 506 ms and the second remained visible until the participant pressed a button. There were 5 blocks of 30 trials (15 novel trials and 15 repetition trials in random order). At the start of each block participants were informed of the target duration for that block: 0.5, 0.75, 1.0, 1.25, or 1.5 seconds. Block order was random and there were no practice trials. Responses shorter than 150 ms or longer than 4000 ms were excluded; their inclusion did not affect the pattern of significant results.

The production task was immediately followed by a surprise old-new recognition test. Twenty five “Old” pictures (encountered during the production task) were randomly intermixed with 25 “New” pictures which had not been seen before. Participants made button presses to indicate whether or not each picture had been seen earlier in the experiment. The Old pictures were always items which had been shown as the first member of each pair on non-repetition trials of the production task. Thus, they were images which had been seen exactly once for 506 ms. Participants were not told in advance that they would complete a memory test, and Old pictures were never drawn from the final block of the production task, ensuring a substantial lag between “study” and “test”. The set of Old pictures was randomly determined for each participant. The events on each trial were: blank screen for 882–1059 ms; stimulus for 506 ms, blank screen until response.

## References

[1] Church RM, Gibbon J (1982). Temporal generalization.. Journal of Experimental Psychology: Animal Behavior Processes.

[2] Rosen S (1992). Temporal information in speech: Acoustic, auditory and linguistic aspects.. Philosophical Transactions of the Royal Society B-Biological Sciences.

[3] Hauser M (1996). The Evolution of Communication..

[4] Buratto LG, Matthews WJ, Lamberts K (2009). When are moving images remembered better? Study–test congruence and the dynamic superiority effect.. Quarterly Journal of Experimental Psychology.

[5] Matthews WJ, Benjamin C, Osborne C (2007). Memory for moving and static images.. Psychonomic Bulletin & Review.

[6] Stone JV (1998). Object recognition using spatiotemporal signatures.. Vision Research.

[7] Lander K, Davies R (2007). Exploring the role of characteristic motion when learning new faces.. Quarterly Journal of Experimental Psychology.

[8] Goldstone S, Lhamon WT, Sechzer J (1978). Light intensity and judged duration.. Bulletin of the Psychonomic Society.

[9] Xuan B, Zhang D, He S, Chen X (2007). Larger stimuli are judged to last longer.. Journal of Vision.

[10] Matthews WJ, Stewart N, Wearden JH (2011). Stimulus intensity and the perception of duration.. Journal of Experimental Psychology: Human Perception and Performance.

[11] Matthews WJ (2011). How do changes in speed affect the perception of duration?. Journal of Experimental Psychology: Human Perception and Performance.

[12] Brown SW (1995). Time, change, and motion: The effects of stimulus movement on temporal perception.. Perception & Psychophysics.

[13] Beckmann JS, Young ME (2009). Stimulus dynamics and temporal discrimination: Implications for pacemakers.. Journal of Experimental Psychology: Animal Behavior Processes.

[14] Wearden JH, Edwards H, Fakhri M, Percival A (1998). Why “Sounds are judged longer than lights”: Application of a model of the internal clock in humans.. Quarterly Journal of Experimental Psychology.

[15] Goldstone S, Lhamon WT (1974). Studies of auditory-visual differences in human time judgments: 1. Sounds are judged longer than lights.. Perceptual and Motor Skills.

[16] Jones LA, Poliakoff E, Wells J (2009). Good vibrations: Human interval timing in the vibrotactile modality.. Quarterly Journal of Experimental Psychology.

[17] Gibbon J, Church RM, Meck WH, Gibbon J, Allan L (1984). Scalar timing in memory.. Annals of the New York Academy of Sciences, Volume 423: Timing and time perception.

[18] Treisman M (1963). Temporal discrimination and the indifference interval: implications for a model of the ‘internal clock’.. Psychological Monographs.

[19] Killeen PR, Weiss NA (1987). Optimal timing and the Weber fraction.. Psychological Review.

[20] Thomas EAC, Weaver WB (1975). Cognitive processing and time perception.. Perception & Psychophysics.

[21] Zakay D, Block RA (1997). Temporal cognition.. Current Directions in Psychological Science.

[22] Eagleman DM, Pariyadath V (2009). Is subjective duration a signature of coding efficiency?. Philosophical Transactions of the Royal Society B-Biological Sciences.

[23] Mauk MD, Buonomano DV (2004). The neural basis of temporal processing.. Annual Review of Neuroscience.

[24] Tse PU, Intriligator J, Rivest J, Cavanagh P (2004). Attention and the subjective expansion of time.. Perception & Psychophysics.

[25] Pariyadath V, Eagleman DM (2007). The effect of predictability on subjective duration.. PLoS ONE.

[26] Seifried T, Ulrich R (2010). Does the asymmetry effect inflate the temporal expansion of odd stimuli?. Psychological Research.

[27] Pariyadath V, Eagleman DM (2008). Brief subjective durations contract with repetition.. Journal of Vision.

[28] Grill-Spector K, Henson R, Martin A (2006). Repetition and the brain: neural models of stimulus specific effects.. Trends in Cognitive Sciences.

[29] Henson RNA, Rugg MD (2003). Neural response suppression, haemodynamic repetition effects, and behavioural priming.. Neuropsychologia.

[30] Ulrich R, Nitschke J, Rammsayer T (2006). Perceived duration of expected and unexpected stimuli.. Psychological Research.

[31] Jones LA, Wearden JH (2003). More is not necessarily better: Examining the nature of the temporal reference memory component in timing.. Quarterly Journal of Experimental Psychology.

[32] Witherspoon DA, Allan LG (1985). The effect of a prior presentation on temporal judgments in a perceptual identification task.. Memory & Cognition.

[33] Ono F, Kawahara JI (2008). The effect of false memory on temporal perception.. Psychological Research.

[34] Hancock PA, Rausch R (2010). The effects of age, sex, and interval duration on the perception of time.. Acta Psychologica.

[35] Danckert JA, Allman A-AA (2005). Time flies when you're having fun: Temporal estimation and the experience of boredom.. Brain and Cognition.

[36] Faul F, Erdfelder E, Lang A-G, Buchner A (2007). G*Power 3: A flexible statistical power analysis program for the social, behavioral, and biomedical sciences.. Behavior Research Methods.

[37] Brown SW (1997). Attentional resources in timing: Interference effects in concurrent temporal and nontemporal working memory tasks.. Perception & Psychophysics.

[38] Penton-Voak IS, Edwards H, Percival A, Wearden JH (1996). Speeding up an internal clock in humans? Effects of click trains on subjective duration.. Journal of Experimental Psychology: Animal Behavior Processes.

[39] Zakay D (1993). Time estimation methods – do they influence prospective duration estimates?. Perception.

[40] Matthews WJ (2011). Can we use verbal estimation to dissect the internal clock? Differentiating the effects of pacemaker rate, switch latencies, and judgment processes.. Behavioural Processes.

[41] Eagleman DM (2008). Human time perception and its illusions.. Current Opinion in Neurobiology.

[42] Noguchi Y, Kakigi R (2006). Time representations can be made from nontemporal information in the brain: An MEG study.. Cerebral Cortex.

[43] Henson RN, Rylands A, Ross E, Vuilleumeir P, Rugg MD (2004). The effect of repetition lag on electrophysiological and haemodynamic correlates of visual object priming.. Neuroimage.

[44] Ono F, Kawahara J (2005). The effect of unconscious priming on temporal production.. Consciousness and Cognition.

[45] Kahneman D, Frederick S, Gilovich T, Griffin D, Kahneman D (2002). Representativeness revisited: Attribute substitution in intuitive judgment.. Heuristics and Biases: The Psychology of Intuitive Judgment.

[46] Droit-Volet S (2010). Stop using time reproduction tasks in a comparative perspective without further analyses of the role of the motor response: The example of children.. European Journal of Cognitive Psychology.

[47] Wearden JH (1999). “Beyond the fields we know…”: Exploring and developing scalar timing theory.. Behavioural Processes.

[48] Forster KI, Forster JC (2003). DMDX: A windows display program with millisecond accuracy.. Behavior Research Methods, Instruments, & Computers.

[49] R Development Core Team (2010). http://www.R-project.org.

[50] Harrell FE (2009). http://CRAN.R-project.org/package=Design.

[51] Loftus GR, Masson MEJ (1994). Using confidence intervals in within-subject designs.. Psychonomic Bulletin & Review.

